# AGING-RELATED HUMAN MONOCYTE TRANSCRIPTOMIC PATHWAYS PREDICT WORSENING MULTIMORBIDITY

**DOI:** 10.1093/geroni/igac059.852

**Published:** 2022-12-20

**Authors:** Jingzhong Ding, Kurt Lohman, Alain Bertoni, Steven Shea, Wendy Post, James Pankow, Stephen Kritchevsky, Yongmei Liu

**Affiliations:** Wake Forest University, Winston Salem, North Carolina, United States; Duke University School of Medicine, Durham, North Carolina, United States; Wake Forest School of Medicine, Winston-Salem, North Carolina, United States; Columbia University, New York, New York, United States; Johns Hopkins University, Baltimore, Maryland, United States; University of Minnesota, Minneapolis, Minnesota, United States; Wake Forest School of Medicine, Winston-Salem, North Carolina, United States; Duke University School of Medicine, Durham, North Carolina, United States

## Abstract

In a cross-sectional analysis, we identified aging-associated monocyte transcriptional modules associated with the multimorbidity in the Multi-Ethnic Study of Atherosclerosis (MESA) study. Here we examine whether these modules predict worsening multimorbidity over time. Transcriptomic profiles were determined in circulating monocytes from 1,264 MESA participants aged 55-94 (51% female, 53% minority). The multimorbidity index was defined as the number of prevalent diseases: cardiovascular disease, type-2 diabetes, hypertension, cancer, dementia, chronic kidney disease, chronic obstructive pulmonary disease, and hip fracture. The mean index at baseline was 1.0 (33%, 45%, and 22% with 0, 1, and 2-6 diseases respectively). At baseline, the index was associated with 5 co-expressed transcriptional modules (FDR< 0.05). During a 6-year follow-up, 449 individuals developed new morbidities. In Cox proportional hazards regression models, 4 (p< 0.05) of these 5 modules including those enriched for downregulation of apoptosis (RH=1.11 per SD increment, p=0.03) and upregulation of complement subcomponent C1q (RH=1.14, p=0.005) predicted incident morbid diseases after adjusting for age, sex, race/ethnicity, study site, and the baseline multimorbidity index. Persons having above the median expression of both modules developed an average of 0.63 new diseases, while those with both below the median developed an average of 0.40 new diseases. These two modules predicted increasing morbidities independently of one another and IL6 levels (p< 0.05). In conclusion, transcriptomic analysis of human immune cells provided evidence that decreased apoptosis and increased immune response predict the onset of a variety of age-related diseases. Whether targeting these pathways will change morbidity risk remains to be demonstrated.

